# Association between triglyceride-glucose and triglyceride-glucose related indices with all-cause mortality in depression participants: a cohort study from NHANES

**DOI:** 10.3389/fpsyt.2025.1614421

**Published:** 2025-07-10

**Authors:** Xinxing Wang, Chengya Feng, Bo Zhang, Guosong Jiang

**Affiliations:** ^1^ Clinical Laboratory, Chengdu BOE Hospital, Chengdu, Sichuan, China; ^2^ Laboratory Department, Deyang Jingyang Maternal and Child Health Hospital, Deyang, Sichuan, China; ^3^ Department of Pulmonary and Critical Care Medicine, The First People’s Hospital of Zhaotong City & The Zhaotong Affiliated Hospital of Kunming Medical University, Zhaotong, Yunnan, China

**Keywords:** all-cause mortality, depression, triglyceride glucose, triglyceride glucose body mass index, triglyceride glucose-waist circumference, triglyceride glucose-waist height ratio, National Health and Nutrition Examination Survey (NHANES)

## Abstract

**Background:**

While the triglyceride-glucose (TyG) index and related indices have been recognized as markers of insulin resistance and cardiometabolic disorders, few studies have examined their association with all-cause mortality in individuals with depression. This study aimed to investigate the relationship between the TyG index, its related indices, and all-cause mortality among patients with depression in the United States.

**Methods:**

A total of 3,179 patients with depression were identified from the National Health and Nutrition Examination Survey (NHANES, 2005–2018). Participants were categorized into tertiles (T1, T2, T3) based on the TyG index and its derived indices: TyG combined with body mass index (TyG-BMI), waist circumference (TyG-WC), and waist-to-height ratio (TyG-WHtR). Cox regression analysis and Kaplan–Meier curve analysis were used to explore the relationship between the independent variable TyG and its derived indicators and the dependent variable all-cause mortality.Curve fitting and threshold effect analyses were performed to evaluate potential nonlinear or dose-response relationships. Subgroup and sensitivity analyses were conducted to validate the robustness of the results.

**Results:**

Over a 13-year follow-up period, both the lowest and highest tertiles of the TyG index and its related indices were associated with significantly increased risks of all-cause mortality compared to the middle tertile. Restricted cubic spline analysis revealed U-shaped nonlinear relationships between these indices and all-cause mortality, with distinct threshold effects. Among the indices, TyG-BMI and TyG-WC demonstrated the strongest associations, though similar trends were observed for the other TyG-related indices.

**Conclusion:**

This study identified nonlinear associations between the TyG index and its related indices (TyG-BMI, TyG-WC, TyG-WHtR) and all-cause mortality in patients with depression, with clear threshold effects. These findings highlight the potential utility of stratified risk assessment and targeted interventions based on these thresholds.

## 1 Introduction

Depression is a globally prevalent psychiatric disorder that significantly impairs quality of life and social functioning. It is associated with elevated all-cause mortality, with individuals experiencing depression having a 2.1-fold higher mortality rate compared to the general population (1, 2). Moreover, depression markedly increases mortality risk through its pathophysiological links to life-threatening comorbidities, such as cardiovascular and cerebrovascular diseases (3–9). Mechanistically, this increased mortality burden may be mediated by insulin resistance (IR), a core feature of metabolic syndrome (MetS) that contributes to multiorgan dysfunction (10). These findings underscore the importance of investigating IR-related biomarkers in the pathophysiology and clinical management of depression.

The triglyceride-glucose (TyG) index has been validated as a practical and cost-effective surrogate marker for IR, demonstrating significant clinical utility in risk stratification and prognostic evaluation across a range of disease states (11, 12). Recent population-based studies have identified strong associations between elevated TyG index levels and all-cause mortality in individuals with cardiometabolic disorders, including cardiovascular disease (11) and diabetes mellitus (9). As a sensitive indicator of systemic metabolic homeostasis, the TyG index has growing relevance in populations where metabolic dysregulation is a major contributor to excess mortality, such as those with depression (12). Further refinement of the TyG index has led to the development of triglyceride-glucose obesity indices, including the TyG-body mass index (TyG-BMI), TyG-waist circumference (TyG-WC), and TyG-waist-to-height ratio (TyG-WHtR). These indices enhance metabolic risk prediction by integrating assessments of IR (via glucose-lipid imbalance), visceral adiposity (reflected by BMI or WC, which indicate adipose tissue dysfunction and increased free fatty acid release), and ectopic lipid deposition (e.g., hepatic steatosis). This multidimensional approach provides a more comprehensive picture of metabolic dysfunction and has demonstrated superior predictive value compared to individual biomarkers (13–16). Based on this evidence, we hypothesize that the TyG index and its related indices are associated with all-cause mortality in individuals with depression.

To explore this hypothesis, we conducted a study using data from the National Health and Nutrition Examination Survey (NHANES) 2005–2018 to examine the associations between TyG indices and all-cause mortality among patients with depression. The findings aim to provide new insights for the prevention and management of depression and to support an integrated care model addressing metabolic-psychiatric multimorbidity.

## 2 Methods

### 2.1 Study design

Analytical datasets were derived from publicly available cycles of the National Health and Nutrition Examination Survey (NHANES) conducted between 2005 and 2018. NHANES utilizes a stratified, multistage probability sampling design to systematically collect nutritional and health data from a nationally representative sample of non-institutionalized U.S. civilians. Ethical approval for all NHANES protocols was granted by the National Center for Health Statistics (NCHS) Ethics Review Board in accordance with the Declaration of Helsinki. Written informed consent was obtained from all participants prior to data collection (17). Detailed documentation regarding NHANES methodology, including sampling procedures and ethical oversight, is available through the Centers for Disease Control and Prevention (CDC) website (https://www.cdc.gov/nchs/nhanes/index.htm).

### 2.2 Study population

**Figure 1:**
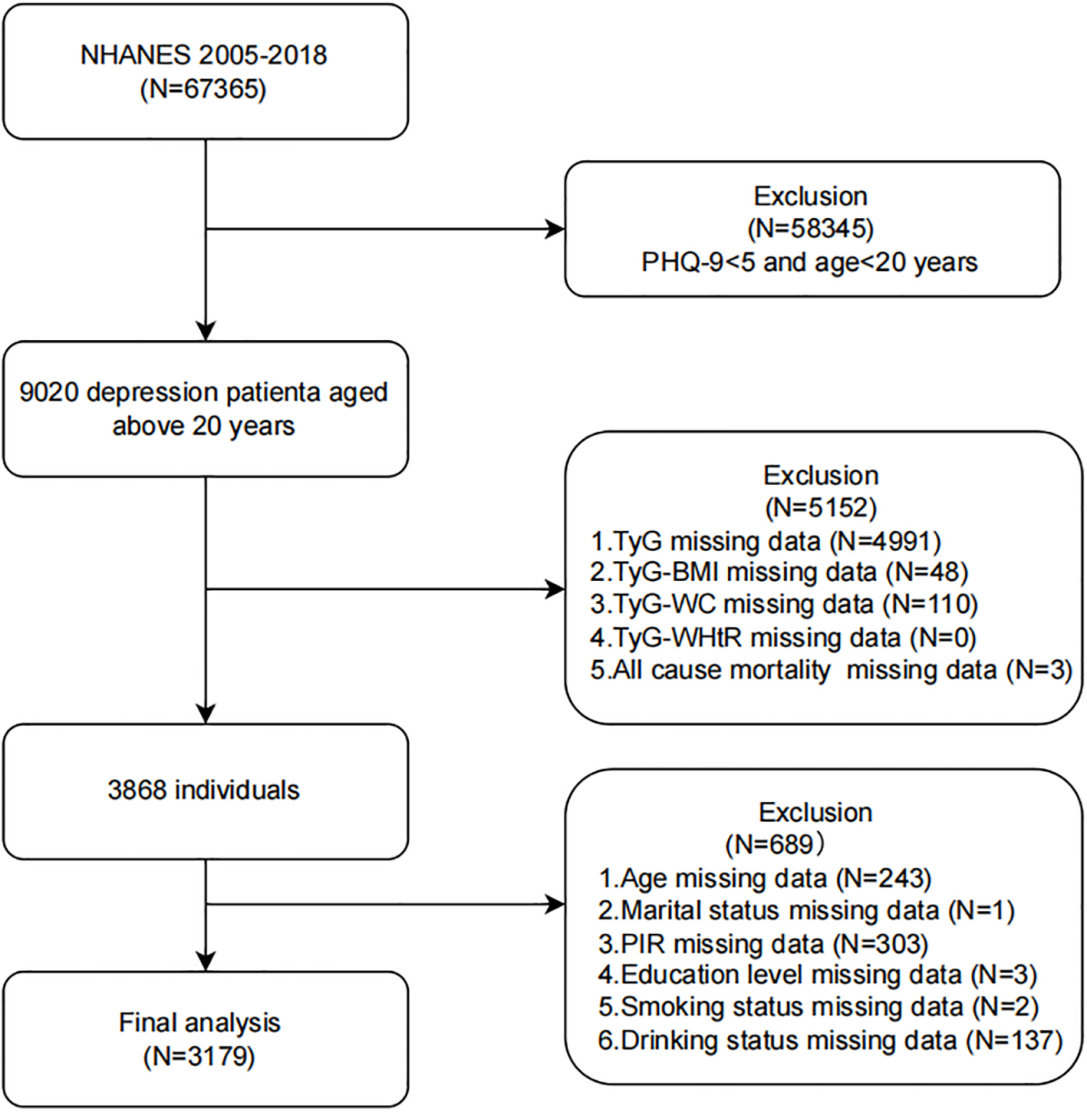
Flow chart of the study population inclusion. Flow chart of participants selection. NHANES, National Health and Nutrition Examination Survey; TyG, triglyceride-glucose; TyG-BMI, TyG with body mass index; TyG-WC, TyG with waist circumference; TyG-WHtR, TyG with waist-to-height ratio; PIR, poverty-income ratio.

As illustrated in **Figure 1**, a total of 67,365 NHANES participants were initially screened. After applying exclusion criteria, 3,179 participants were included in the final analysis. Exclusions were made as follows: 1) individuals aged <20 years and with a Patient Health Questionnaire-9 (PHQ-9) total score ≤5 (18), *N* = 58345; 2) participants with incomplete data on TyG indices and all-cause mortality, *N* = 5152; 3) participants missing covariate data (age, marital status, poverty-income ratio (PIR), educational level, smoking status, or hyperlipidemia), *N* = 689.

### 2.3 TyG and TyG-related indices

Venous blood samples were obtained from fasting participants by trained medical professionals following standardized protocols. These samples were analyzed in a central laboratory to measure serum TG and fasting plasma glucose (FPG) levels using an enzymatic colorimetric method. The TyG index was calculated using the following formula: TyG = ln (TG (mg/dL) × FPG (mg/dL)/2). TG concentrations were measured using the Roche Modular P and Cobas 6000 systems, while FPG levels were assessed using the hexokinase method on a Roche/Hitachi Cobas C 501 analyzer (19).

Participants’ height and weight were measured three times using a stadiometer and weighing scale, respectively, with participants barefoot and wearing minimal clothing. WC was also measured three times by trained staff using a flexible tape positioned at the level of the navel. The mean of the three measurements was used for each parameter in subsequent analyses.

BMI was calculated as weight (kg) divided by height in meters squared (m²). The waist-to-height ratio (WHtR) was calculated as WC (cm) divided by height (cm). To enhance the predictive value of the TyG index, it was combined with BMI, WC, and WHtR to generate the composite indices: TyG-BMI, TyG-WC, and TyG-WHtR. These were calculated using the following formulas (20):

TyG-BMI = TyG × BMI
TyG-WC = TyG × WC
TyG-WHtR = TyG × WHtR

Participants were stratified into tertiles based on each TyG-related index, with the second tertile serving as the reference group for all subsequent analyses.

### 2.4 Outcomes and follow-up

The National Death Index is updated every 4 years, with the most recent follow-up data available through December 31, 2019. Accordingly, the follow-up period for each participant was calculated from the date of their initial examination at the Mobile Examination Center (MEC) until either the occurrence of death or the end of the study period (December 31, 2019), whichever came first. Mortality status was determined through probabilistic linkage of the NHANES cohort with the Centers for Disease Control and Prevention’s National Death Index as of December 31, 2019. All-cause mortality was defined as death from any cause, regardless of etiology. Cause-specific mortality categories were classified according to the International Classification of Diseases, Tenth Revision (ICD-10), and included the following: cardiovascular disorders (codes 054-068), malignant neoplasms (019-043), chronic lower respiratory tract diseases (082-086), unintentional traumatic injuries (112-123), cerebrovascular pathologies (070), Alzheimer’s dementia (052), diabetes mellitus (046), influenza-pneumonia complex (076-078), nephropathies including nephritic syndrome and renal degeneration (097-101), along with residual miscellaneous causes (21, 22).

### 2.5 Ascertainment of depression

The Patient Health Questionnaire-9 (PHQ-9) is a validated 9-item screening tool used to assess the severity of depressive symptoms experienced over the preceding 2 weeks (18, 23). Numerous studies have confirmed its reliability and validity as a diagnostic instrument for depression (23, 24), consistent with the criteria for major depressive disorder as outlined in the Diagnostic and Statistical Manual of Mental Disorders, Fourth Edition (DSM-IV). Each of the nine items is scored from 0 to 3, based on the participant’s response: “not at all,” “several days,” “more than half the days,” or “nearly every day.” The total PHQ-9 score ranges from 0 to 27, with scores of 0–4 indicating no depression, and scores ≥5 suggesting the presence of depressive symptoms. Depression severity was further classified as follows: mild (5–9), moderate (10–14), and severe (≥15). These categories have been used in previous studies to investigate associations between depression severity and other clinical or demographic variables (25, 26).

### 2.6 Assessment of covariates

Demographic variables, including age, sex, race/ethnicity, educational level, marital status, and family income, were collected using a computer-assisted personal interview system. Information on lifestyle factors and comorbidities, including smoking (27), alcohol consumption (28), and hyperlipidemia, was gathered. Hyperlipidemia was defined as meeting any of the following criteria: use of lipid-lowering medication, triglycerides ≥150 mg/dL, total cholesterol ≥200 mg/dL, LDL cholesterol ≥130 mg/dL, or HDL cholesterol <40 mg/dL (29). Physical examinations, including measurements of waist circumference, weight, and height, were performed following standardized protocols at mobile examination centers.

### 2.7 Statistical analysis

All statistical analyses were conducted using R version 4.2.2 (http://www.R-project.org, R Foundation) and Free Statistics version 2.2.0. FreeStatistics is a software package that provides intuitive interfaces for common statistical analyses and data visualization. It uses R as the underlying statistical engine, with a graphical user interface (GUI) developed in Python. A P-value of less than 0.05 was considered statistically significant.

The normality of continuous variables was initially assessed using histogram analysis. For variables with a normal distribution, parametric tests were applied, and comparisons among multiple groups were conducted using analysis of variance (ANOVA), with results reported as mean ± standard deviation. For non-normally distributed variables, non-parametric tests were used, specifically the Kruskal–Wallis test for comparisons across multiple groups, with results expressed as median and interquartile range (IQR). Categorical variables were analyzed using non-parametric methods, primarily the Chi-square test, and were reported as percentages within frequency categories.

Cox proportional hazards regression models were used to estimate hazard ratios (HRs) and 95% confidence intervals (CIs) for the associations between TyG, TyG-BMI, TyG-WC, TyG-WHtR, and all-cause mortality. Trends across tertiles were assessed by modeling tertile levels as ordinal variables and evaluating the corresponding *P*-values. The proportional hazards assumption was tested using the Schoenfeld residuals test, with no violations detected. All models were adjusted for age, sex, race/ethnicity, marital status, education level, poverty-income ratio (PIR), smoking status, alcohol consumption, and hyperlipidemia. To address multicollinearity, variance inflation factors (VIFs) were calculated for all covariates, and only variables with VIF < 5 were retained in the models. Missing covariates were directly removed as they accounted for less than 5% of the dataset. Restricted cubic splines were used to visualize the dose-response relationships between TyG indices and all-cause mortality. The number of knots (three) was selected based on minimizing the Akaike Information Criterion (AIC) to balance model fit and the risk of overfitting (30). In cases of nonlinear associations, potential threshold inflection points were identified, and two-piece Cox proportional hazards models were constructed to examine the relationship on either side of the threshold. Kaplan–Meier survival curves were plotted to illustrate differences in survival rates across tertiles of TyG, TyG-BMI, TyG-WC, and TyG-WHtR among participants with depression.

To further explore the associations between TyG,TyG-related indices and all-cause mortality, subgroup analyses were conducted by stratifying participants according to age, sex, and hyperlipidemia status. In addition, given the hormonal fluctuations associated with menopause and their potential impact on metabolic outcomes, participants were categorized into premenopausal (<45 years), perimenopausal (45–55 years), and postmenopausal (>55 years) groups for further stratified analysis in fully adjusted models (31, 32). Sensitivity analyses were performed to test the robustness of the results by: 1) excluding the lowest 0.5% and highest 0.5% of values to reduce the influence of outliers, and 2) excluding participants with diabetes to assess whether the associations between TyG indices and all-cause mortality remained consistent with the primary findings.

## 3 Results

### 3.1 Baseline characteristics of participants

A total of 3,179 participants with depression were included in the analysis (mean age = 48.8 years, SD = 17.1), of whom 1,941 (61.1%) were female. During a mean follow-up period of 7.16 years, 360 deaths (11.3%) from all causes were recorded. **Table 1** presents the baseline demographic and clinical characteristics of participants stratified by TyG index tertiles. Participants in the higher TyG tertiles were more likely to be older, non-Hispanic White, consume alcohol more frequently, and have a significantly higher prevalence of hyperlipidemia (*P* < 0.01).

**Table 1:** Characteristics of the study participants by baseline TyG index in NHANES 2005-2018.

Variables	Total (*N* = 3179)	T1 *(N* = 1059)	T2 (*N* = 1059)	T3 (*N* = 1061)	*P*-value
		6.79-8.38	8.38-8.98	8.98-12.85	
Age,mean(SD), years	48.8 ± 17.1	43.8 ± 17.4	49.9 ± 17.4	52.8 ± 15.3	< 0.001
Sex, n (%)					< 0.001
Male	1238 (38.9)	344 (32.5)	407 (38.4)	487 (45.9)	
Female	1941 (61.1)	715 (67.5)	652 (61.6)	574 (54.1)	
Race, n (%)					< 0.001
Non-Hispanic White	1432 (45.0)	413 (39)	485 (45.8)	534 (50.3)	
Non-Hispanic Black	660 (20.8)	338 (31.9)	189 (17.8)	133 (12.5)	
Mexican American	488 (15.4)	116 (11)	175 (16.5)	197 (18.6)	
Other Hispanic	337 (10.6)	94 (8.9)	124 (11.7)	119 (11.2)	
Other Race	262 (8.2)	98 (9.3)	86 (8.1)	78 (7.4)	
Marital status, n (%)					0.003
No	1529 (48.1)	554 (52.3)	494 (46.6)	481 (45.3)	
Yes	1650 (51.9)	505 (47.7)	565 (53.4)	580 (54.7)	
PIR, n (%)					0.150
<1.30	1343 (42.2)	432 (40.8)	445 (42.0)	466 (43.9)	
1.31-3.50	1183 (37.2)	384 (36.3)	401 (37.9)	398 (37.5)	
≥3.50	653 (20.5)	243 (22.9)	213 (20.1)	197 (18.6)	
Education level, n (%)					< 0.001
Less than high school	936 (29.4)	264 (24.9)	321 (30.3)	351 (33.1)	
High school or equivalent	785 (24.7)	245 (23.1)	268 (25.3)	272 (25.6)	
Above high school	1458 (45.9)	550 (51.9)	470 (44.4)	438 (41.3)	
Smoking status, n (%)					< 0.001
Never	1510 (47.5)	571 (53.9)	484 (45.7)	455 (42.9)	
Former	740 (23.3)	197 (18.6)	246 (23.2)	297 (28)	
Now	929 (29.2)	291 (27.5)	329 (31.1)	309 (29.1)	
Drinking status, n (%)					< 0.001
Never	412 (13.0)	136 (12.8)	137 (12.9)	139 (13.1)	
Former	592 (18.6)	155 (14.6)	192 (18.1)	245 (23.1)	
Current	2175 (68.4)	768 (72.5)	730 (68.9)	677 (63.8)	
Hyperlipidemia, n (%)	2362 (74.3)	510 (48.2)	817 (77.1)	1035 (97.5)	< 0.001
TyG, Mean (SD)	8.7 ± 0.7	8.0 ± 0.3	8.7 ± 0.2	9.5 ± 0.5	< 0.001
TYG-BMI, Mean (SD)	266.6 ± 75.7	224.0 ± 64.0	267.0 ± 67.1	308.7 ± 70.8	< 0.001
TYG-WC, Mean (SD)	893.7 ± 191.3	755.1 ± 150.3	890.5 ± 147.3	1035.4 ± 162.0	< 0.001
TyG-WHtR, Mean (SD)	5.4 ± 1.2	4.6 ± 0.9	5.4 ± 0.9	6.2 ± 1.0	< 0.001

PIR, poverty-income ratio; TyG, triglyceride-glucose; TyG-BMI, TyG with body mass index; TyG-WC,TyG with waist circumference; TyG-WHtR, TyG with waist-to-height ratio; Other Race, Including Multi-Racial; SD, standard deviation.

### 3.2 Association between TyG-related indices and all-cause mortality

After adjusting for potential confounding factors, a significant curvilinear association was observed between the TyG index, its related indices, and all-cause mortality risk (*P* < 0.05), as shown in **Table 2** (Model 3). Using the T2 group as the reference, the fully adjusted model revealed that the TyG index was significantly associated with mortality risk: the hazard ratio (HR) was 1.34 (95% CI: 1.01–1.79, *P* = 0.041) for the T1 group and 1.33 (95% CI: 1.03–1.72, *P* = 0.030) for the T3 group.

**Table 2:** The association of TyG, TyG-BMI, TyG-WC and TyG-WHtR with All-cause mortality in depression participants.

Variable	Model 1		Model 2		Model 3	
	HR(95%CI)	*P*-value	HR(95%CI)	*P*-value	HR(95%CI)	*P*-value
**TyG**						
T1	1.37 (1.04~1.80)	0.026	1.30 (0.98~1.73)	0.065	1.34 (1.01~1.79)	0.041
T2	1(Ref)		1(Ref)		1(Ref)	
T3	1.35 (1.06~1.72)	0.017	1.27 (0.99~1.63)	0.057	1.33 (1.03~1.72)	0.030
**TYG-BMI**						
T1	1.51 (1.18~1.93)	0.001	1.46 (1.13~1.87)	0.003	1.35 (1.05~1.74)	0.021
T2	1(Ref)		1(Ref)		1(Ref)	
T3	1.21 (0.93~1.57)	0.162	1.12 (0.86~1.46)	0.398	1.13 (0.87~1.47)	0.367
**TYG-WC**						
T1	1.69 (1.29~2.22)	<0.001	1.67 (1.27~2.19)	<0.001	1.60 (1.21~2.11)	0.001
T2	1(Ref)		1(Ref)		1(Ref)	
T3	1.42 (1.11~1.82)	0.006	1.34 (1.05~1.72)	0.020	1.37 (1.07~1.76)	0.014
**TyG-WHtR**						
T1	1.45 (1.11~1.89)	0.006	1.36 (1.04~1.78)	0.024	1.30 (0.99~1.71)	0.056
T2	1(Ref)		1(Ref)		1(Ref)	
T3	1.23 (0.96~1.57)	0.108	1.15 (0.89~1.47)	0.282	1.16 (0.9~1.49)	0.253

TyG, triglyceride-glucose; TyG-BMI, TyG with body mass index; TyG-WC, TyG with waist circumference; TyG-WHtR, TyG with waist-to-height ratio; PIR, poverty-income ratio; HR, hazard ratio.

Model 1: Adjusted for age, sex.

Model 2:Adjusted for Model 1 + race, marital status, PIR, education level.

Model 3: Adjusted for Model2 + smoking status,drinking status, hyperlipidemia.

The TyG-WC index demonstrated a more pronounced dose-response relationship. In the fully adjusted model, the T1 group had the highest mortality risk (HR: 1.60, 95% CI: 1.21–2.11, *P* = 0.001), while the T3 group also showed a significantly elevated risk compared to the reference group (HR: 1.37, 95% CI: 1.07–1.76, *P* = 0.014).

For both the TyG-BMI and TyG-WHtR indices, a significantly increased risk of all-cause mortality was observed in the T1 groups. Specifically, for the TyG-BMI index, the HR was 1.35 (95% CI: 1.05–1.74, *P* = 0.021) in the fully adjusted model. Similarly, for the TyG-WHtR index, the T1 group showed a significantly elevated risk in Model 1 (HR: 1.45, 95% CI: 1.11–1.89, *P* = 0.006) (**Table 2**).

### 3.3 Curve fitting and inflection point analysis of TyG-related indices and all-cause mortality

**Figure 2:**
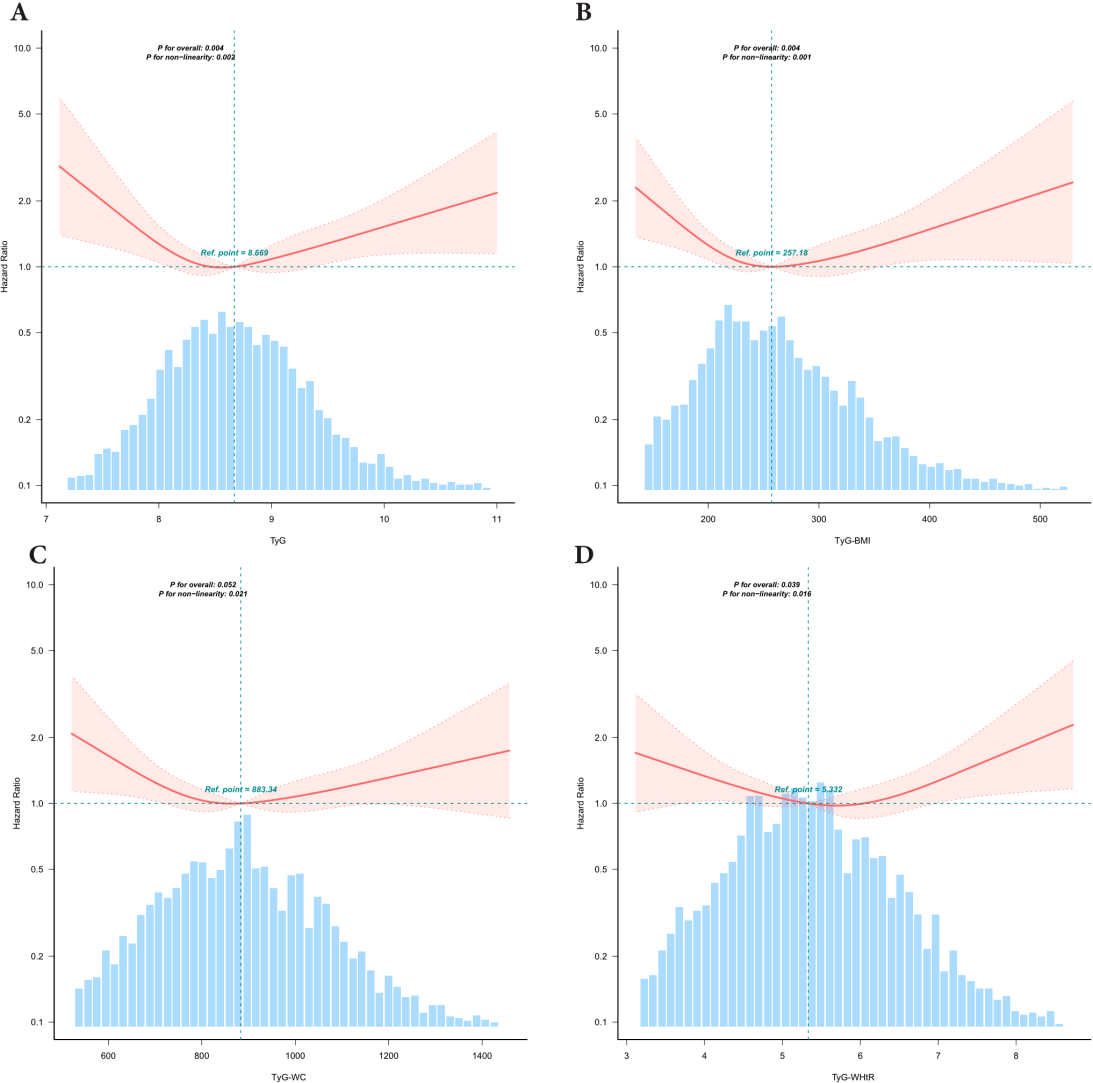
Association between TyG and TyG related Indices with All-Cause Mortality risk in depression participants. TyG,triglyceride-glucose;TyG-BMI,TyG with body mass index;TyG-WC,TyG with waist circumference;TyG-WHtR, TyG with waist-to-height ratio,PIR,poverty-income ratio. Restricted cubic spline curve for the association of TyG-related indices with all-cause mortality. All-cause mortality for **(A)** TyG index, **(B)** TyG-BMI index, **(C)** TyG-WC index and **(D)** TyG-WHtR index. The solid red lines indicate hazard ratios (HRs), with the width of the red shaded areas representing the 95% confidence intervals (95% CIs). The light blue dashed line serves as the reference line, The intersection point of the reference line is the median by default. The light blue shaded bar areas in the background illustrate the population distribution of TyG-related indices. Adjusted for age, sex, race, marital status, PIR, education level, smoking status,drinking status, hyperlipidemia.

After adjusting for potential confounders, we observed U-shaped nonlinear associations between the TyG index and its derivative indices (TyG-BMI, TyG-WC, and TyG-WHtR) and the risk of all-cause mortality (*P* for nonlinearity < 0.05 for all) (**Figure 2**). For the TyG index, inflection point analysis identified a threshold at 8.443. Below this value, each unit increase in TyG was associated with a 59% reduction in mortality risk (HR: 0.409, 95% CI: 0.205–0.817), whereas each unit increase above 8.443 corresponded to a 34% increase in risk (HR: 1.336, 95% CI: 1.039–1.717). Significant threshold effects were also identified for the derivative indices: TyG-BMI (inflection point: 240.848), TyG-WC (835.303), and TyG-WHtR (5.828). The risk patterns showed statistically significant differences before and after each threshold (likelihood ratio test, *P* < 0.01). These nonlinear relationships were further validated through segmented regression modeling (**Table 3**).

**Table 3:** Threshold effect analysis of the relationship of TyG, TyG-BMI, TyG-WC and TyG-WHtR with All-cause mortality in depression participants.

Breakpoint	HR (95%CI)	*P* -value
TyG		
<8.443	0.409 (0.205,0.817)	0.0113
≥8.443	1.336 (1.039,1.717)	0.0237
Likelihood Ratio test	–	0.0040
TyG-BMI		
<240.848	0.9904 (0.9845,0.9963)	0.0014
≥240.848	1.0024 (0.9996,1.0051)	0.0946
Likelihood Ratio test	–	<0.001
TyG-WC		
<835.303	0.9974 (0.9951,0.9997)	0.0239
≥835.303	1.0008 (0.9998,1.0018)	0.1347
Likelihood Ratio test	–	0.0020
TyG-WHtR		
<5.828	0.832 (0.667,1.038)	0.1035
≥5.828	1.352 (1.021,1.791)	0.0353
Likelihood Ratio test	–	0.0040

TyG, triglyceride-glucose; TyG-BMI, TyG with body mass index; TyG-WC, TyG with waist circumference; TyG-WHtR, TyG with waist-to-height ratio, PIR, poverty-income ratio.

Adjusted for age, sex, race, marital status, PIR, education level, smoking status, drinking status, and hyperlipidemia. HR, hazard ratio.

### 3.4 Subgroup analyses of TyG-related indices and all-cause mortality

**Figure 3:**
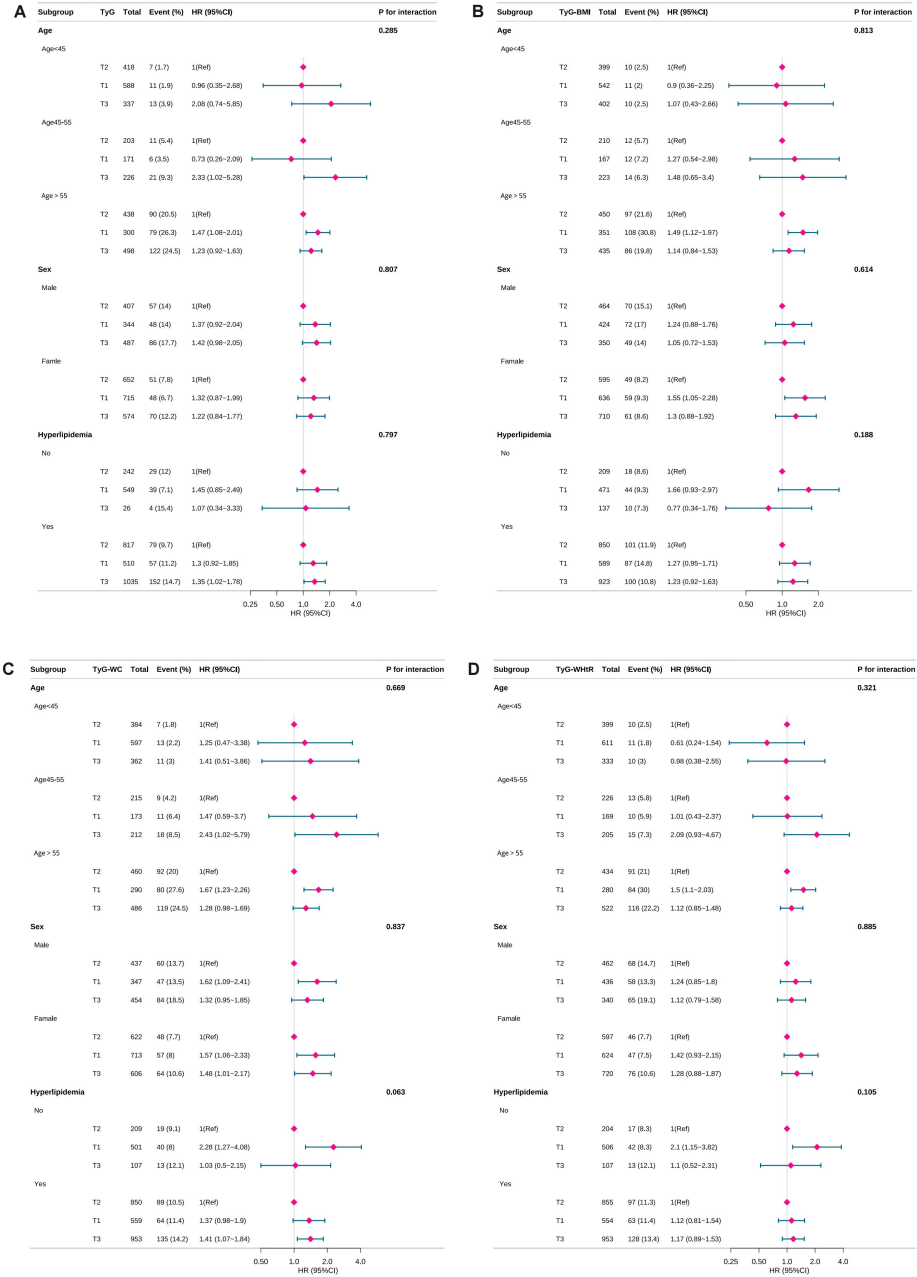
Subgroup analyses of TyG-related indices and all-cause mortality. TyG, triglyceride-glucose; TyG-BMI, TyG with body mass index; TyG-WC, TyG with waist circumference; TyG-WHtR, TyG with waist-to-height ratio, PIR, poverty-income ratio. HR, hazard ratio. Forest plots showing the association of TyG and TyG-related indices with the risk of all-cause mortality in depressed individuals. All-cause mortality for **(A)** TyG index, **(B)** TyG-BMI index, **(C)** TyG-WC index and **(D)** TyG-WHtR index. Adjusted for age, sex, race, marital status, PIR, education level, smoking status, drinking status, hyperlipidemia. Purple diamonds indicate point estimates of effect sizes, The length of the line segment indicates the 95% confidence interval of the effect size.

Subgroup analyses were performed to assess whether the associations between TyG,TyG-related indices and all-cause mortality varied across demographic or clinical subgroups. No significant interaction effects were detected for any of the three indices, indicating that the observed associations were consistent across subgroups. These findings confirm the robustness of the results. Subgroup analysis results are presented in **Figure 3**.

### 3.5 Kaplan–Meier survival curves of TyG-related indices and all-cause mortality

Kaplan–Meier survival analyses demonstrated that TyG and its related indices were significantly associated with all-cause mortality in patients with depression (*P* < 0.001; **Supplementary Figure S1**). Participants in the T3 group had a markedly lower survival rate compared to those in the T1 group, indicating that higher levels of TyG and TyG-related indices are associated with increased mortality risk.

### 3.6 Sensitivity analysis

Sensitivity analyses were conducted to further validate the robustness of the findings. First, extreme values were trimmed by retaining data within the 0.5%–99.5% percentiles. Second, participants with diabetes were excluded from the analysis. The results remained consistent across both approaches, thereby reinforcing the stability and reliability of the primary associations (**Supplementary Tables S1**, **S2**).

## 4 Discussion

Our study yields four key findings. First, the TyG index and TyG-related indices demonstrated a strong association with all-cause mortality in individuals with depression. This association remained significant after adjusting for covariates and showed no interaction effects across age, sex, or hyperlipidemia subgroups. Second, the relationship between TyG indices and mortality exhibited a U-shaped nonlinear pattern with clear threshold effects. Third, Kaplan–Meier survival curves confirmed that higher TyG and TyG-related indices were associated with increased all-cause mortality risk. Fourth, these associations remained robust in sensitivity analyses, including the exclusion of outliers and diabetic participants, thereby reinforcing the reliability of our conclusions.

Although the TyG index has been linked to all-cause mortality across various disease states, limited research has explored its role in predicting mortality among individuals with depression. Most existing studies focus on the association between TyG and depressive symptoms, typically within cross-sectional designs that preclude causal inference (33–35). In contrast, our study leveraged a large, population-based cohort (2005–2018) and employed a longitudinal approach. Prior to conducting Cox regression analyses, we rigorously tested the proportional hazards assumption and adjusted for multiple covariates. The results provide compelling evidence of a significant association between TyG levels and mortality risk in depressed populations, addressing a notable gap in the literature.

The observed U-shaped association between TyG indices and all-cause mortality may reflect dual pathophysiological mechanisms (11). At lower TyG levels (T1), impaired energy homeostasis (36) and neuroendocrine dysregulation may worsen depressive symptoms and increase metabolic vulnerability. In contrast, higher TyG levels (T3) likely represent advanced IR (12), systemic inflammation, and cardiometabolic comorbidities—well-established contributors to increased mortality in mood disorders (5). Notably, the TyG-WC index exhibited the most pronounced U-shaped curve, suggesting that central adiposity may intensify both ends of this metabolic paradox (11, 37). From a clinical perspective, these findings highlight the need for individualized glycemic monitoring thresholds in patients with depression, moving beyond simplistic linear risk models (11). Therapeutic strategies should aim to balance metabolic regulation with adequate nutritional support, particularly given depression’s complex relationship with both hyperphagia and appetite loss (5, 38).

The association between TyG-related indices and mortality risk may be mediated by multiple physiological mechanisms (39). First, the notably high risk observed for TyG-WC in the non-hyperlipidemia subgroup (HR: 2.28) suggests that visceral fat accumulation may directly impair vascular endothelial function through the release of free fatty acids (40). This effect may be further exacerbated by the chronic inflammatory state commonly seen in depression, characterized by elevated levels of inflammatory cytokines such as IL-6 and TNF-α, which contribute to oxidative stress (41, 42). Second, threshold effect analysis revealed a protective association below the TyG index inflection point of 8.443 (HR: 0.409), possibly reflecting the role of moderate insulin sensitivity in maintaining metabolic homeostasis (43, 44). In contrast, values above this threshold may contribute to the accumulation of glucolipotoxicity, resulting in mitochondrial dysfunction and the formation of advanced glycation end-products (AGEs), both of which are implicated in increased mortality risk (41, 45).

Although the inflection point for TyG-WC (835.303) did not reach statistical significance (*P* = 0.1347), the consistently elevated risk in the T1 group of the segmented model (HR: 1.58, *P* = 0.001) supports the “visceral fat–inflammation axis” theory (46). This theory posits that pro-inflammatory factors secreted by visceral adipose tissue may act synergistically with IR to accelerate the development of atherosclerosis (47).

This study defined depression using a validated computational approach widely adopted in NHANES and prior research, rather than formal clinical diagnostic criteria. Specifically, the Patient Health Questionnaire-9 (PHQ-9) was used as a screening tool. While the PHQ-9 is a widely used and validated instrument with acceptable sensitivity and specificity for identifying depressive symptoms in both primary care and research settings, it is not a substitute for a comprehensive clinical diagnosis. Clinical diagnosis typically requires an in-depth interview, assessment of symptom duration and functional impairment, and exclusion of alternative medical or psychiatric conditions, elements that the PHQ-9 may not fully capture. Several additional limitations warrant consideration. First, the absence of hormonal data required the use of chronological age to define menopausal status, which limits the precision of our interpretations regarding associations between TyG/TyG-related indices and all-cause mortality in the depressed cohort. Second, the relatively advanced age of many participants presents a limitation, as causes of death (e.g., suicide vs. cardiovascular events) likely vary substantially across age groups. Third, although we performed comprehensive multivariate adjustments for demographic, metabolic, and lifestyle factors, the possibility of residual confounding cannot be ruled out. Additionally, while the exclusion of participants with incomplete data may have introduced selection bias, multiple sensitivity analyses supported the robustness of our primary findings. To improve precision in future studies, we recommend incorporating standardized clinical assessments for participant inclusion.

The findings of this study offer novel evidence linking IR to mortality risk in individuals with depression, supporting the rationale for IR-targeted interventions in the clinical management of this population. Furthermore, we demonstrated a U-shaped association between the TyG index and all-cause mortality, with both low and high TyG values associated with increased mortality risk. These findings support the development of threshold-based, stratified interventions, enabling clinicians to tailor therapeutic strategies according to individual TyG index profiles. Overall, the evidence presented here contributes to advancing precision medicine approaches for managing metabolic–psychiatric comorbidities.

## 5 Conclusion

The TyG index and its related indices are significantly associated with all-cause mortality in patients with depression, with evidence of threshold effects. Based on these findings, it is recommended that TyG-BMI and TyG-WC be incorporated into routine clinical risk assessments for individuals with depression. Early interventions—such as lifestyle modifications or targeted anti-inflammatory treatments—may be especially beneficial for individuals without hyperlipidemia. Furthermore, the development of multidimensional risk prediction models that integrate metabolic, psychological, and social factors may advance precision medicine in the management of depression–metabolic comorbidities.

## Data Availability

The original contributions presented in the study are included in the article/**Supplementary Material**. Further inquiries can be directed to the corresponding author.
