# Supplementary material for: Association between triglyceride-glucose and triglyceride-glucose related indices with all-cause mortality in depression participants: a cohort study from NHANES
Source: Front Psychiatry. 2025 Jul 10;16:1614421. doi: 10.3389/fpsyt.2025.1614421 (PMC12290894; doi:10.3389/fpsyt.2025.1614421)
Supplement: Supplementary file 1 [file SupplementaryFile1.docx]

**Supplementary Material**

| **Variable** | **Model 1** |  |  | **Model 2** |  |  | **Model 3** |  |
| --- | --- | --- | --- | --- | --- | --- | --- | --- |
|  | **HR(95%)** | ***P*-value** |  | **HR(95%)** | ***P*-value** |  | **HR(95%)** | ***P*-value** |
| **TyG** |  |  |  |  |  |  |  |  |
| T1 | 1.37 (1.04~1.81) | 0.024 |  | 1.28 (0.97~1.7) | 0.081 |  | 1.33 (1~1.77) | 0.046 |
| T2 | 1(Ref) |  |  | 1(Ref) |  |  | 1(Ref) |  |
| T3 | 1.34 (1.05~1.72) | 0.019 |  | 1.28 (0.97~1.7) | 0.081 |  | 1.35 (1.04~1.75) | 0.023 |
| **TYG-BMI** |  |  |  |  |  |  |  |  |
| T1 | 1.51 (1.18~1.93) | 0.001 |  | 1.45 (1.13~1.86) | 0.004 |  | 1.34 (1.04~1.73) | 0.023 |
| T2 | 1(Ref) |  |  | 1(Ref) |  |  | 1(Ref) |  |
| T3 | 1.2 (0.92~1.56) | 0.185 |  | 1.12 (0.86~1.46) | 0.395 |  | 1.12 (0.86~1.47) | 0.388 |
| **TYG-WC** |  |  |  |  |  |  |  |  |
| T1 | 1.7 (1.29~2.23) | <0.001 |  | 1.64 (1.25~2.16) | <0.001 |  | 1.58 (1.19~2.08) | 0.001 |
| T2 | 1(Ref) |  |  | 1(Ref) |  |  | 1(Ref) |  |
| T3 | 1.42 (1.11~1.82) | 0.006 |  | 1.34 (1.04~1.72) | 0.023 |  | 1.41 (1.1~1.81) | 0.007 |
| **TyG-WHtR** |  |  |  |  |  |  |  |  |
| T1 | 1.45 (1.11~1.9) | 0.006 |  | 1.36 (1.04~1.78) | 0.024 |  | 1.31 (0.99~1.71) | 0.055 |
| T2 | 1(Ref) |  |  | 1(Ref) |  |  | 1(Ref) |  |
| T3 | 1.22 (0.95~1.57) | 0.115 |  | 1.16 (0.9~1.49) | 0.255 |  | 1.16 (0.9~1.49) | 0.247 |

**Table S1.Sensitivity analysis: Association of TyG, TyG-BMI, TyG-WC, and TyG-WHtR with all-cause mortality in participants with depression (only 99.5% of the data is displayed).**

TyG ,triglyceride-glucose;TyG-BMI,TyG with body mass index;TyG-WC,TyG with waist circumference ;TyG-WHtR, TyG with waist-to-height ratio;PIR,poverty-income ratio;HR, hazard ratio.

Model 1: Adjusted for age, sex

Model 2 :Adjusted for Model 1 + race, marital status, PIR , education level

Model 3: Adjusted for Model2 + smoking status,drinking status,hyperlipidemia

**Table S2. Sensitivity Analysis: Association of TyG, TyG-BMI, TyG-WC, and TyG-WHtR with All-Cause Mortality in Participants with Depression (Excluding the Influence of Diabetic Patients).**

| **Variable** | **Model 1** |  |  | **Model 2** |  |  | **Model 3** |  |
| --- | --- | --- | --- | --- | --- | --- | --- | --- |
|  | **HR(95%)** | ***P*-value** |  | **HR(95%)** | ***P*-value** |  | **HR(95%)** | ***P*-value** |
| **TyG** |  |  |  |  |  |  |  |  |
| T1 | 1.42 (1.02~1.97) | 0.035 |  | 1.27 (0.9~1.79) | 0.167 |  | 1.30(0.92~1.84) | 0.133 |
| T2 | 1(Ref) |  |  | 1(Ref) |  |  | 1(Ref) |  |
| T3 | 1.15 (0.8~1.65) | 0.464 |  | 1.28 (0.97~1.7) | 0.081 |  | 1.11 (0.76~1.62) | 0.590 |
| **TYG-BMI** |  |  |  |  |  |  |  |  |
| T1 | 1.67 (1.21~2.29) | 0.002 |  | 1.61 (1.17~2.22) | 0.004 |  | 1.45 (1.04~2.01) | 0.027 |
| T2 | 1(Ref) |  |  | 1(Ref) |  |  | 1(Ref) |  |
| T3 | 1.03 (0.68~1.57) | 0.879 |  | 0.98 (0.64~1.49) | 0.928 |  | 0.96 (0.63~1.46) | 0.841 |
| **TYG-WC** |  |  |  |  |  |  |  |  |
| T1 | 1.83 (1.3~2.56) | <0.001 |  | 1.77 (1.25~2.49) | 0.001 |  | 1.65 (1.17~2.33) | 0.005 |
| T2 | 1(Ref) |  |  | 1(Ref) |  |  | 1(Ref) |  |
| T3 | 1.39 (0.97~2) | 0.076 |  | 1.29 (0.89~1.86) | 0.178 |  | 1.33 (0.92~1.92) | 0.126 |
| **TyG-WHtR** |  |  |  |  |  |  |  |  |
| T1 | 1.6 (1.15~2.23) | 0.005 |  | 1.52 (1.09~2.12) | 0.014 |  | 1.4 (1~1.97) | 0.049 |
| T2 | 1(Ref) |  |  | 1(Ref) |  |  | 1(Ref) |  |
| T3 | 1.24 (0.85~1.79) | 0.264 |  | 1.14 (0.79~1.66) | 0.481 |  | 1.14 (0.78~1.66) | 0.494 |

TyG ,triglyceride-glucose;TyG-BMI,TyG with body mass index;TyG-WC,TyG with waist circumference ;TyG-WHtR, TyG with waist-to-height ratio;PIR,poverty-income ratio;HR, hazard ratio.

Model 1: Adjusted for age, sex

Model 2 :Adjusted for Model 1 + race, marital status, PIR , education level

Model 3: Adjusted for Model2 + smoking status,drinking status,hyperlipidemia

**Fig.S1. The Kaplan-Meier survival curves for TyG index and its derived indices and all-cause mortality**

**
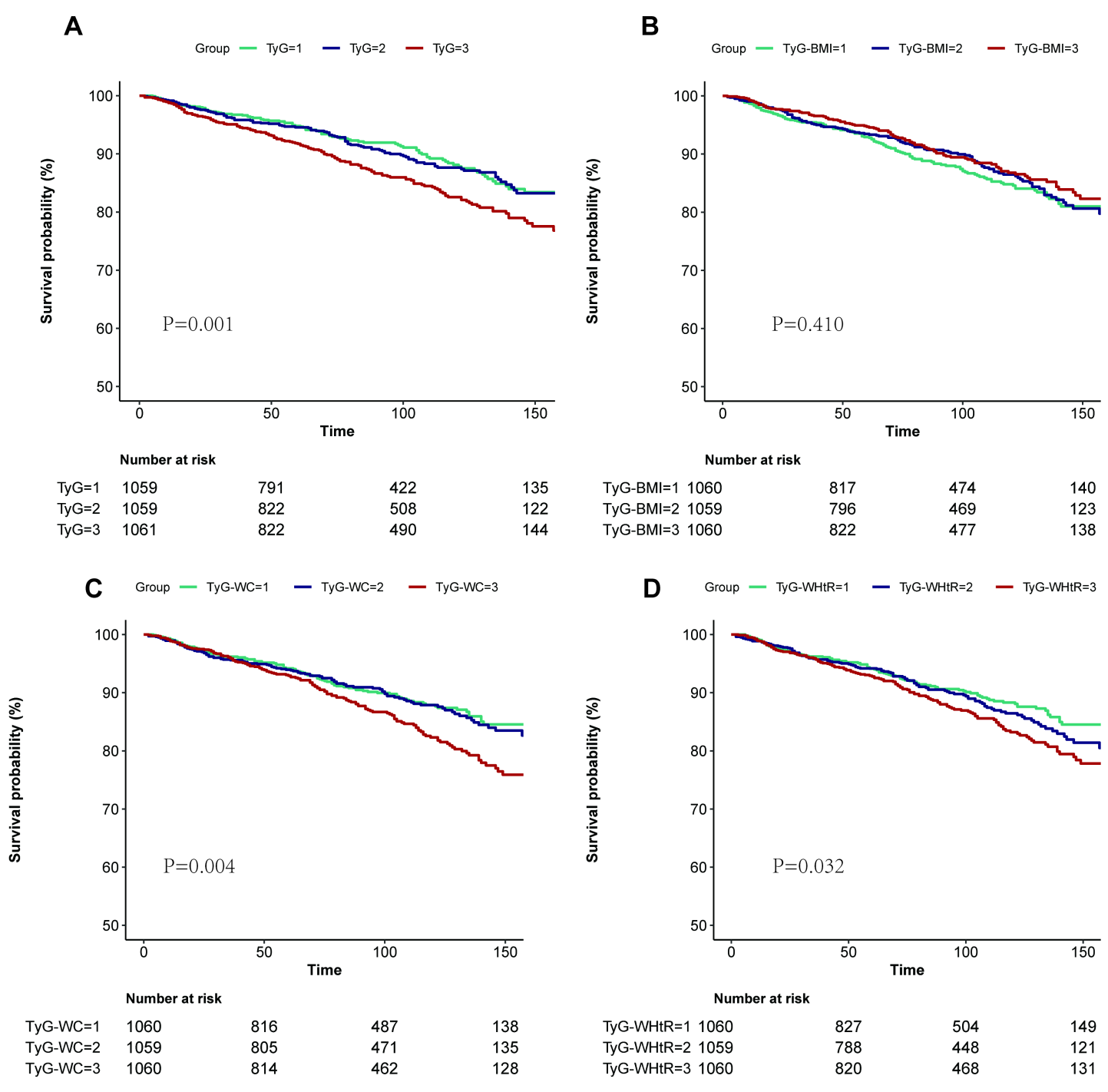
**

The Kaplan-Meier survival curves for all-cause mortality (A, B, C, and D) among the TyG index and its derivatives. TyG, Triglyceride‒glucose; TyG-BMI,TyG with body mass index;TyG-WC,TyG with waist circumference ;TyG-WHtR, TyG with waist-to-height ratio;P,P value.
